# Association of Alzheimer’s disease risk variants on the *PICALM* gene with PICALM expression, core biomarkers, and feature neurodegeneration

**DOI:** 10.18632/aging.103814

**Published:** 2020-11-07

**Authors:** Wei Xu, Chen-Chen Tan, Xi-Peng Cao, Lan Tan, for the Alzheimer’s Disease Neuroimaging Initiative

**Affiliations:** 1 Department of Neurology, Qingdao Municipal Hospital, Qingdao University, Qingdao, China; 2 Clinical Research Center, Qingdao Municipal Hospital, Qingdao, China

**Keywords:** PICALM, Alzheimer’s disease, expression, biomarker, neurodegeneration

## Abstract

It is still unclear how *PICALM* mutations influence the risk of Alzheimer’s disease (AD). We tested the association of AD risk variants on the *PICALM* gene with PICALM expression and AD feature endophenotypes. Bioinformatic methods were used to annotate the functionalities and to select the tag single nucleotide polymorphisms (SNPs). Multiple regressions were used to examine the cross-sectional and longitudinal influences of tag SNPs on cerebrospinal fluid (CSF) AD biomarkers and neurodegenerations. A total of 59 SNPs, among which 75% were reported in Caucasians, were associated with AD risk. Of these, 73% were linked to PICALM expression in the whole blood (p < 0.0001) and/or brain regions (p < 0.05). Eleven SNPs were selected as tag SNPs in Caucasians. rs510566 (T allele) was associated with decreased CSF ptau and ptau/abeta42 ratio. The G allele of rs1237999 and rs510566 was linked with greater reserve capacities of the hippocampus, parahippocampus, middle temporal lobe, posterior cingulate, and precuneus. The longitudinal analyses revealed four loci that could predict dynamic changes of CSF ptau and ptau/abeta42 ratio (rs10501610, p = 0.0001) or AD feature neurodegeneration (rs3851179, rs592297, and rs7480193, p < 0.005). Overall, the genetic, bioinformatic, and association studies tagged four SNPs (rs3851179, rs7480193, rs510566, and rs1237999) as the most prominent *PICALM* loci contributing to AD in Caucasians.

## INTRODUCTION

Alzheimer’s disease (AD) is a common neurodegenerative disease with a complex etiology underpinned by genetic elements [1]. In 2009, the phosphatidylinositol binding clathrin assembly protein (*PICALM*) gene was linked to the risk of AD by a genome-wide association study (GWAS) [2]. In 2019, two large, independent GWASs reported that *PICALM* ranked third in influencing AD risk [3, 4], further reinforcing the importance of *PICALM* as a genetic contributor. Moreover, the causal relationship between *PICALM* mutations and AD has been supported by multiple lines of evidence in the past decade [5]. First, the association of *PICALM* variations with AD risk was constantly replicated in different populations [6]. Also, PICALM expression was found altered in AD compared to controls [7]. The potential functionalities of relevant variations were investigated, and several intergenic loci (e.g., rs3851179 [8] and rs588076 [9]) were linked to PICALM expression. In addition, the mechanisms by which PICALM or *PICALM* variations influence AD risk were also widely explored. The, *in vivo* and *in vitro* studies have suggested that PICALM might be involved in clearing AD core pathologies (including β-amyloid (Aβ) [10] and tauopathy [11]) and accelerating synaptic loss [5]. We also reported several *PICALM* loci associated with greater reserve capacities of posterior cingulate in non-demented elderly [12]. Nonetheless, the identified risk loci were numerous and inconsistent across various studies, possibly due to ethnic heterogeneity or an insufficient sample size.

We hypothesized that if a risk genetic locus could contribute to AD, it would: 1) be associated with AD risk, 2) influence the protein (this being PICALM) expression, and 3) modulate AD endophenotypes, such as core pathologies or neurodegeneration. Here, we aimed to combine the evidence from genetic, bioinformatic, and association studies to provide a comprehensive framework of the relationships of the *PICALM* gene with AD. We summarized the *PICALM* susceptibility loci and tested the influences of the tag SNPs on PICALM expression, cerebrospinal fluid (CSF) AD biomarkers, and AD feature neurodegenerations.

## RESULTS

### AD susceptibility loci profile of the *PICALM* gene

### Search results and study characteristics

**Figure 1:**
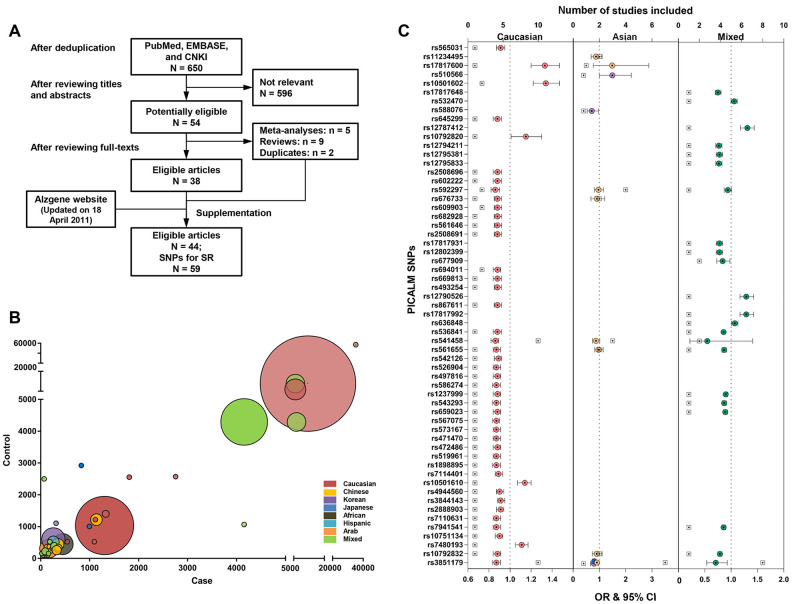
**Constructing the profile for Alzheimer’s disease risk variants of the *PICALM* gene via systematic review and meta-analysis.** The flow chart of the literature selection: Finally, a total of 44 case-control studies were included, with 59 loci within or near *PICALM* gene associated with AD risk (**A**). The majority of included studies had a small-to-moderate sample size < 5000 (mainly from Asian, African, and Hispanic populations) and and the minority had a larger sample from Caucasian population or mixed-race population based on multi-center organizations (**B**). Associations of *PICALM* variations with AD risk in different populations (**C**).

Figure 1A shows the flow chart of the systematic review. The search yielded 650 studies after any duplicates were removed. Previous systematic reviews, the Alzgene website, and the reference list of the studies found were also reviewed. In total, 44 case-control studies that linked 59 loci within or near the *PICALM* gene to AD risk were included. Supplementary Table 1 shows the detailed characteristics of the included studies, of which the majority (91%) had a small-to-moderate sample size < 5000 (mainly from Asian, African, and Hispanic populations), and some had large samples consisting of Caucasian or mixed-race populations (Figure 1B and Supplementary Table 2). Females were well-represented in both the case and control groups (mostly > 50%). 27% of the studies provided pathological evidence for AD diagnosis, and 68% conducted their analysis after matching or adjusting for essential covariates. The quality of included studies was moderate (median score = 6.5, interquartile range [IQR] = 2, Supplementary Table 3).

### PICALM loci associated with AD risk

A total of 59 loci, consisting of 31 within the *PICALM* gene and 28 downstream of the gene, were associated with AD risk in various ethnicities, including 45 (76%) in Caucasians, 2 in Chinese (rs3851179 and rs541458), 3 in Koreans (rs3851179, rs588076, and rs510566), 1 in Japanese (rs3851179), and 21 (36%) in mixed-race populations (Figure 1C). Three loci (rs3851179, rs541458, and rs592297) were further explored using a meta-analysis. rs3851179 (allele A) was associated with lower AD risk, with the effect size ranging from 9% to 29% in Caucasians (I^2^ = 38%), Chinese (I^2^ = 42%), Japanese, Koreans, and mixed-race populations (I^2^ = 38%) (Supplementary Figure 1). rs541458 was revealed as an AD risk locus in Caucasians (OR = 0.86, 95% CI = 0.83 to 0.89, I^2^ = 9.8%) and Chinese (OR = 0.87, 95% CI = 0.74 to 1.02, I^2^ = 0%). rs592297 tended to be a risk locus in Caucasians (OR = 0.96, 95% CI = 0.91 to 1.02, I^2^ = 0%), but not Chinese (OR = 0.97, 95% CI = 0.81 to 1.16, I^2^ = 66%) (Figure 1C). The analysis of rs3851179 and rs541458 in Caucasians were further examined for publication bias (n ≥ 10) and no bias was revealed.

### Functional annotations and tag SNPs

The enhancer enrichment analysis showed that the abovementioned *PICALM* variants were significantly enriched in specific brain regions (e.g., middle hippocampus, inferior temporal lobe, prefrontal lobe, and substantia nigra) and blood cells (e.g., primary monocytes) (Supplementary Table 4), suggesting that these variants might be linked to the regulation of gene expression in these places. To validate and characterize these associations, expression analyses were performed to determine whether PICALM expression levels could be influenced by these variants. Consistently, the eQTL analyses showed that 73% of these variations could significantly regulate PICALM expression in the whole blood (6 loci, p < 0.0001) and abovementioned brain regions (40 loci, p < 0.05; Supplementary Table 5). Finally, to further test the association of PICALM variations with AD endophenotypes, 11 SNPs were selected by LD analysis. These loci could independently capture 100% of all alleles at r^2^ ≥ 0.8 (Supplementary Figure 2).

### Associations of tag SNPs with AD endophenotypes

### Participant characteristics

A total of 712 (203 CN, 395 MCI, and 114 AD) and 877 (275 CN, 461 MCI, and 141 AD) individuals were included in the association analyses of CSF AD biomarkers and neurodegeneration, respectively. The mean age of the study sample was 73.9 years and females accounted for roughly 42%. Compared with those free of dementia, individuals with AD tended to be older, less educated, and *APOE*4 carriers. (Table 1).

**Table 1:** Summary of characteristics of ADNI sample.

**Baseline diagnosis**	**CSF AD biomarker**			**P value**	**Neurodegeneration**			**P value**
	**Total**	**AD**	**Non-demented**		Total*	**AD**	**Non-demented**	
n	712	114	598	…	877	141	736	…
Age, mean ± SD	73.9 ± 7.3	75.0 ± 8.5	73.6 ± 7.0	0.02	73.9 ± 7.0	75.0 ± 8.1	73.7 ± 6.7	0.04
Male/Female, n	416/296	67/47	349/249	0.94	515/362	81/60	434/302	0.74
Education, mean ± SD	15.9 ± 2.8	15.3 ± 3.0	16.0 ± 2.7	0.04	15.8 ± 2.8	15.0 ± 3.0	16.0 ± 2.8	0.0003
*APOE*_Ɛ_4 carrier, %	44% (314)	66.7% (76)	40% (238)	< 0.0001	47% (412)	70% (99)	43% (313)	< 0.0001

Abbreviations: CSF = Cerebrospinal fluid; AD = Alzheimer’s disease; SD = Standard deviation.

The significance of difference between the two diagnostic groups was examined by two-sample t test or Mann-Whitney U test when appropriate (for continuous variable) and Pearson's Chi-squared test with continuity correction (for categorical variable).

*The sample size is 614 for analyses of specific brain regions (parahippocampal region, posterior cingulate, and precuneus). The characteristics pattern is similar with that in Table 1 (see Supplementary Table 6).

### Association of PICALM tag SNPs with AD biomarkers

**Figure 2:**
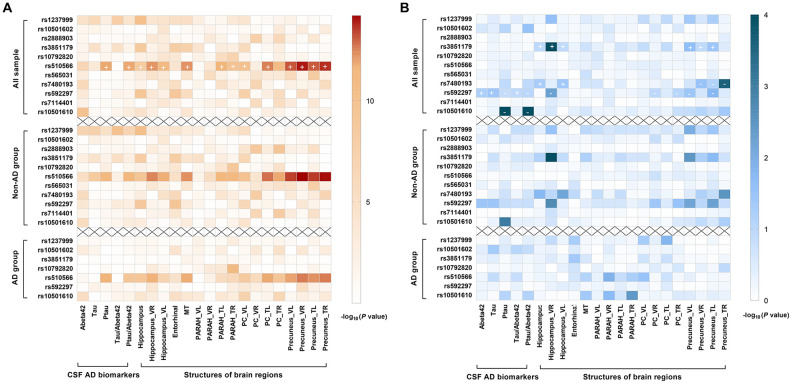
**Summary results for the cross-sectional (2A) and longitudinal (2B) relationships of *PICALM* variations with CSF AD biomarkers and AD feature neurodegeneration, stratified by clinical diagnosis.** After Bonferroni correction, rs510566 (G allele) was associated with lower CSF Aβ42, higher ptau, higher ptau/ Aβ42 ratio, and neurodegeneration in five feature regions, including HIPPO, PARAH, MT, PC and PRE (**A**). Longitudinally, rs10501610 (T allele) was associated with a slower rise in ptau and ptau/Aβ42 ratio. The same trends were also found for rs592297 and rs3851179, though the associations did not survive the Bonferroni correction. rs3851179 (G allele), rs592297 (C allele), and rs7480193 (G allele), were significantly associated with a faster rate of hippocampal atrophy. Similar trends were found for PRE and PC regions, but the p values failed to reach statistical significance after Bonferroni correction. (**B**) The “+” represent the beta-value is positive while “-” indicates the beta-value is negative.

**Figure 3:**
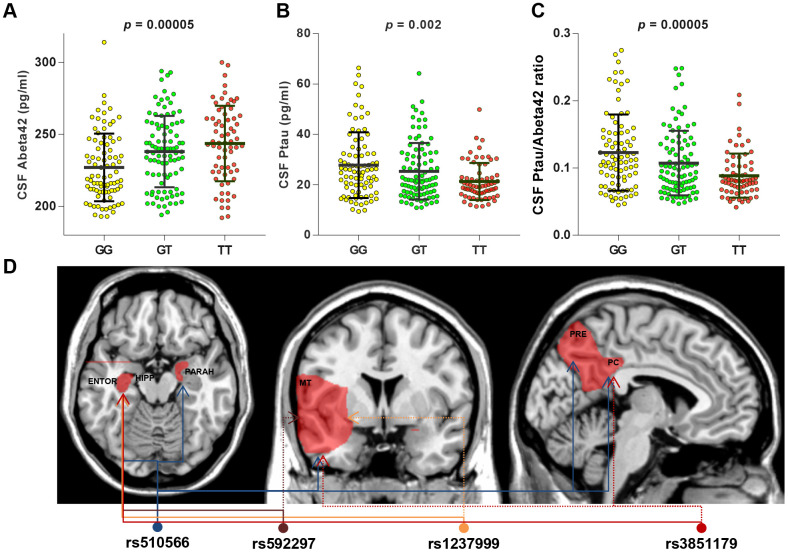
**CSF AD biomarkers and distribution of brain region affected by specific *PICALM* variations in subgroup analyses.** T allele of rs510566 had associations with higher CSF levels of Abeta42 (**A**) and lower CSF levels of ptau (**B**) or ptau/abeta42 (**C**), which remained significant only in A (-) subgroup. In addition, the associations with specific loci showed significant trends in *APOE*4 (-) subgroup, including rs1237999 (HIPPO, ENTOR, and MT), rs592297 (HIPPO, ENTOR, and MT), and rs3851179 (HIPPO, ENTOR, MT, and PC) (**D**).

After Bonferroni correction, two loci showed significant associations with baseline levels of CSF AD biomarkers. After adjusting for age, gender, education, *APOE*4 status, and clinical diagnosis, rs510566 (G allele) was associated with lower levels of CSF Aβ42 (p = 0.048) as well as higher levels of ptau (p = 0.0006) and the ptau/Aβ42 ratio (p = 0.0006) (Figure 2A). These associations were not influenced by subgrouping according to clinical diagnosis (Figure 2A) and *APOE*4 status (Supplementary Figure 3). However, the association of rs510566 remained significant only in the A (-) subgroup (Figure 3A–3C). In addition, we found nominally significant or suggestive associations of rs1237999 (A allele) with lower levels of CSF Aβ42 (p = 0.042) as well as higher levels of tau (p = 0.030), ptau (p = 0.011), and the ptau/Aβ42 ratio (p = 0.045) in the non-demented population (Figure 2A).

Longitudinal analyses showed that the T allele of rs10501610 was associated with a slower rise in ptau and the ptau/Aβ42 ratio (SNP × time interaction p = 0.0001). The same trends were also found for rs592297 and rs3851179 (p < 0.05), for which the associations did not survive Bonferroni correction (Figure 2B).

### Associations of PICALM tag SNPs with AD feature neurodegeneration

Similarly, rs510566 was significantly associated with neurodegeneration in five feature regions, including HIPPO (p < 0.0001), PARAH (p < 0.0045), MT (p < 0.0001), PC (p < 0.0001) and PRE (p < 0.0001) (Figure 2A). The above associations remained significant in the non-demented population but not in those living with AD. In addition, the associations with specific loci showed suggestive significance in the *APOE*4 (-) subgroup, including rs1237999 (p = 0.002 for HIPPO, p = 0.009 for ENTOR, and p = 0.018 for MT), rs592297 (p = 0.003 for HIPPO, p = 0.008 for ENTOR, and p = 0.014 for MT), and rs3851179 (p = 0.005 for HIPPO, p = 0.0037 for ENTOR, p < 0.05 for MT, and p < 0.05 for PC; Figure 3D).

Longitudinal analyses revealed that three loci, including rs3851179 (G allele), rs592297 (C allele), and rs7480193 (G allele), were significantly associated with a faster rate of hippocampal atrophy (p < 0.0045). Similar trends were found for the PRE and PC regions, but the p values failed to reach statistical significance after Bonferroni correction. (Figure 2B)

### Multiple evidence-based summary

**Figure 4:**
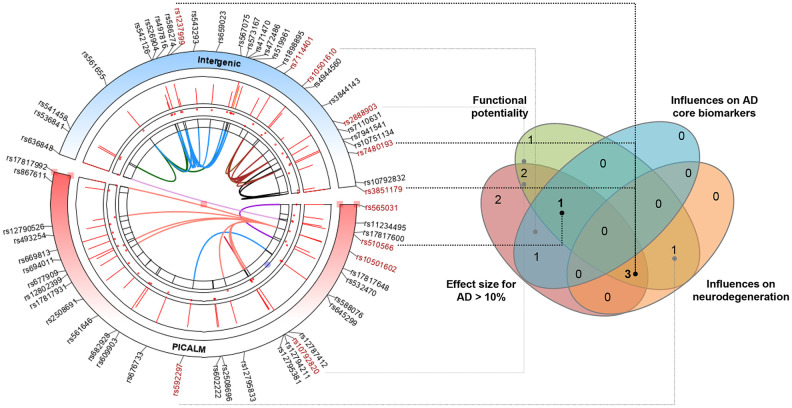
**Identifying the most prominent PICALM variations contributing to AD based on multiple-evidence summary.** Finally, we integrated lines of evidence, including the effect size of association with AD risk, potential functionality, and the influences on AD feature endophenotype (CSF biomarkers or neurodegeneration), to identify the *PICALM* loci with high credibility of evidence to support their relationships with AD. Among the 11 tag variations in the Caucasian population, four (rs3851179, rs7480193, rs510566, and rs1237999) were highlighted by the overlapping sources of evidence.

Finally, we combined evidence, including the effect size of association with AD risk, potential functionality, and the influence on AD feature endophenotype (CSF biomarker or neurodegeneration), to identify the *PICALM* loci with a high credibility of evidence to support the relationships with AD. Among the 11 tag variations in the Caucasian population, four (rs3851179, rs7480193, rs510566, and rs1237999) were highlighted by overlapping sources of evidence (Figure 4).

## DISCUSSION

The current study provided consolidated evidence that the associations of four loci in the *PICALM* gene with AD were robust in the Caucasian population. The overlapping characteristics of these variations were that they 1) rendered a prominent risk difference for AD, 2) could influence PICALM expression in the brain or blood, and 3) were associated with feature biomarkers or neurodegeneration of AD.

Among the four identified variations, three were located in the intergenic region at 5′ to the *PICALM* gene. rs3851179, a transcription factor (TF) binding site, was the first AD risk locus of *PICALM* [13]. Its association with AD risk has been widely studied over the past decade. As revealed by our study, the A allele was associated with a 9% to 29% reduced risk of AD in a number of ethnicities. However, the sample size used was small in ethnicities other than Caucasians (Supplementary Figure 1). The location of rs3851179, which was situated in the promoter flanking region (PFR), has indicated its potential functionality. It has been proven that rs3851179 could influence total PICALM expression in multiple brain regions [8], such as the cingulate cortex and medulla. We further found that protective A allele was associated with slower atrophy rates of the hippocampus, middle temporal lobe, and precuneus. This might help explain previous findings of an association of rs3851179 with information processing speed [14] and cognitive impairment [15]. In addition to structural changes, it was also found that rs3851179 was linked to impairments in the functional connectivity of the hippocampus [16] and default mode network (DMN), which are observed in AD [17]. All these findings strengthen the functional role of rs3851179 in AD.

rs7480193 was also a transcription factor (TF) binding site at 5′ to the *PICALM* gene. The G allele renders an 11% increased risk of AD, which might be explained by the following two reasons. 1) rs7481093 could influence PICALM expression in specific brain regions, and 2) the G allele of rs7481093 was associated with faster hippocampal atrophy. As a CCCTC binding factor in a regulatory region, rs1237999 (G allele) was associated with a lower AD risk. Notably, this variation achieved a high functional score (Regulome DB Score = 2a), and it could modulate PICALM expression in multiple brain regions. We further demonstrated that the protective G allele was also associated with higher CSF abeta42 levels, lower CSF tau levels, and greater hippocampal volume. Though little is known about the influence of rs1237999 on cognitive decline, it has been shown that rs561655, a locus in high LD with rs1237999, could significantly influence the onset age of AD.

Situated in the intron area, rs510566 (G allele) was linked to a higher risk of AD, and it could influence PICALM expression in the frontal cortex. There have been few investigations on the influence of this variation on AD-related phenotypes. We found a G allele-dependent relationship between rs510566 and higher levels of CSF ptau or ptau/abeta42, which might suggest rs510566 could modulate the metabolism of core AD pathologies. Nonetheless, we also found unexpected protective effects of the G allele on brain reserve capabilities. However, no significant effects were found for both features in the longitudinal analyses. Additional studies are required to explain this phenomenon.

*PICALM* variations might be closely linked to the structural basis and functional activity of the DMN [12], which is impaired in the early stage of AD [18, 19]. Here, we showed that the brain structure could be influenced by *PICALM* variations, and cerebral PICALM expression was altered predominately in the precuneus, posterior cingulate, hippocampus, and frontal cortex, all of which are pivotal components of DMN. These findings further strengthen our hypothesis.

This study had several limitations. The sample sizes in the analyses of several tag loci or brain regions were relatively small (Supplementary Table 5), which might reduce the statistical power. The influence of PICALM loci on AD feature endophenotypes was restricted to Caucasians, and the generalizability to other ethnicities warrants further investigation. The current study focused on the common variations of the *PICALM* gene and did not consider any rare but highly functional mutations. Finally, we did not provide direct evidence regarding the mechanism of the selected loci. The functionalities warrant further investigation with *in vivo* or *in vitro* studies.

In summary, we identified several variations as the most prominent *PICALM* loci contributing to AD in the Caucasian population. Our work lay a foundation to test functionalities of these loci and to explore the genetic mechanisms how *PICALM* contribute to AD.

## MATERIALS AND METHODS

### AD susceptibility loci profile of the *PICALM* gene

The AD susceptibility loci profile of the *PICALM* gene was constructed using a systematic review and meta-analysis. The detailed methods of this can be found in Appendix 1. In brief, we systematically searched electronic databases for articles published in English (PubMed and EMBASE) and Chinese (CNKI) using the terms “PICALM” and “CALM” till Jan 11, 2020. Literature that reported associations of the *PICALM* gene with AD risk were included. The Newcastle-Ottawa Quality Assessment Scale (NOS) was used to evaluate the quality of eligible studies. When a loci was reported by ≥3 studies in the same race, its corresponding multivariable-adjusted odds ratios and 95% confidence intervals (CI) were log-transformed and pooled using random models (DerSimonian-Laird method) [20]. Heterogeneity was assessed using the Q test and quantified by the *I^2^* metric.

### Functional annotations of the loci

Bioinformatics was employed for the functional annotations of *PICALM* loci associated with AD risk. Specifically, the single nucleotide polymorphism (SNP) annotations were performed using the NCBI Database of Single Nucleotide Polymorphisms (dbSNP, GRCh38.p12) (http://www.ncbi.nlm.nih.gov/SNP/) [21] and SNP and CNV Annotation Database (SCAN) (http://www.scandb.org/newinterface/index_v1.html). The potential regulatory functions were examined using HaploReg v4.1 [22], RegulomeDBv1.1 (http://regulome.stanford.edu/) [23], and the 1000 Genomes Project (http://www.internationalgenome.org/). Linkage disequilibrium analyses were conducted based on data from the 1000 Genomes Project (EUR).

HaploReg v4.1 was employed to conduct the enhancer enrichment analysis to evaluate in which cell types the tag variants were significantly enriched. Enhancers were defined using the Roadmap Epigenomics data [24]. A binomial test was performed using all 1000 genome variants with a MAF > 5% in any population as the background set. A total of 28 blood cells and 13 brain cells were selected for the analyses. To test whether the tag SNPs could affect PICALM expression, expression quantitative trait loci (eQTL) analyses were performed using multiple publicly available datasets on human brain tissues (including UK Brain Expression Consortium (https://ukbec.wordpress.com/) and MayoeGWAS study (https://www.synapse.org/#!Synapse:syn3157225) [25]) and the whole blood (including Blood eQTL browser [26], Consortium for the Architecture of Gene Expression browser [27], NCBI Molecular QTL Browser Search database [28], and Framingham Heart Study eQTL database [28]). In addition, the Variant Effect Predictor (http://asia.ensembl.org/Homo_sapiens/Tools/VEP?db=core;expand_form=true;tl=WVCEGgWKR01l7Xcl-6022877) and Alzdata (www.alzdata.org) [29] were also used for the functional annotation of the tag SNPs.

### Association of the tag SNPs with CSF AD biomarkers and neurodegeneration

### Study participants

Data used in this section were obtained from the Alzheimer’s Disease Neuroimaging Initiative (ADNI) database (adni.loni.usc.edu). We confined our analysis to non-Hispanic white individuals because all selected SNPs were reported in Caucasians. As a multicenter study, ADNI is designed to develop clinical, imaging, genetic, and biochemical biomarkers for the early detection and tracking of AD. The participants were adults aged 55-90 years with normal cognition (NC), mild cognitive impairment (MCI), or mild Alzheimer’s disease. Further information can be found at http://www.adni-info.org/ and in previous reports [30–32]. ADNI was approved by the institutional review boards of all participating institutions, and written informed consent was obtained from all participants or their guardians according to the Declaration of Helsinki.

### Measurements of CSF AD biomarkers

CSF procedural protocols have been described previously [13]. In brief, CSF was collected by lumbar puncture in 10 ml polypropylene tubes before being sent to the lab within 2 hours. The samples were centrifuged at 2000g for 10 minutes. The thaw/freezing cycle was limited so as not to surpass 2 times. CSF Aβ42, tau, and ptau_181_ were measured using the INNOBIA AlzBio3 immunoassay (Fujirebio, Belgium). The within-batch precision values were <10% for="" Aβ<sub="">1-42, t-tau and ptau181 (5.1-7.8%, 4.4-9.8% and 5.1-8.8%, respectively).

### MRI measurement

The process of MRI acquisition in ADNI has been described elsewhere [33]. In brief, ADNI MRIs were acquired at multiple sites with 1.5T GE, Philips, and Siemens MRI scanners using the magnetization prepared rapid acquisition gradient echo (MP-RAGE) sequence. Two high-resolution T1-weighted MRI scans were collected for each participant using a sagittal 3D MP-RAGE sequence with an approximate TR = 2400 ms, minimum full TE, approximate TI = 1000 ms, and approximate flip angle of 8 degrees. Scans were obtained with a 24 cm field of view and an acquisition matrix of 192 x 192 x 166 (x, y, z dimensions) to yield a standard voxel size of 1.25 x 1.25 x 1.2 mm. Images were then reconstructed to give a 256 x 256 x 166 matrix with a voxel size of approximately 1 x 1 x 1.2 mm.

Herein, six brain regions, including the hippocampus (HIPPO), parahippocampal region (PARAH), entorhinal cortex (ENTOR), middle temporal lobe (MT), posterior cingulate (PC), and precuneus (PRE), were selected as regions of interest (ROIs), because these regions were specifically affected by AD [34–38]. A total of 877 (16% AD) individuals were included for analyses of HIPPO, ENTOR and MT, and 614 (16% AD) were included for other regions.

### Genotyping

The sequencing data in ADNI-1 and ADNI-GO/2 was downloaded. In ADNI-1, GenomeStudio v2009.1 (Illumina) was used to process the array data. In ADNI-GO/2, loci were genotyped by the Human OmniExpress BeadChip (Illumina, Inc, San Diego, CA). The ADNI-1 and ADNI-GO/2 datasets consisted of 620,901 and 710,618 genotyped variants respectively, both of which included rs7412 and rs429358 used to define the *APOEε2/ε3/ε4* isoforms as previously described [39]. Finally, all tag SNPs were genotyped in ADNI-GO/2. All but four loci (rs2888903, rs565031, rs7480193, and rs7114401) were genotyped in ADNI-1.

### Statistical analyses

R version 3.5.1, GraphPad Prism 7.00, and TBtools software were used for the statistical analyses and figure preparation. A Bonferroni-corrected p-value of < 0.0045 (11 tag SNPs) was considered statistically significant. Chi-square tests (for categorical variables), a one-way analysis of variance (ANOVA; for continuous variables with normal distributions), and the Kruskal-Wallis test (for continuous variables with skewed distributions) were used to compare the baseline demographic, clinical, and diagnostic characteristics.

In the case of skewed distribution (Shapiro-Wilk test > 0.05) for the dependent variable, a transformation was performed to approximate a normal distribution via the “car” package of R software. Linear regressions were used to explore the cross-sectional associations of tag SNPs (additive model) with AD endophenotypes, including CSF levels of AD biomarkers (Aβ42 and tau) and volume/thickness of ROI. Covariates included age, gender, education, *APOE4* status, diagnosis, and intracranial volume (ICV) at baseline. T-tau and P-tau181 were expressed in ratio to Aβ42 because they were reported as better predictors of cerebral β-amyloid deposition [40, 41] and cognitive decline [42, 43] than themselves alone. We re-ran all analyses according to the baseline diagnosis (AD vs. non-demented), *APOE4* status (positive vs. negative), and Aβ pathological status (A positive vs. A negative) by A-T-N criteria, in which A positive was defined as positive evidence of cerebral Aβ deposition defined by positron emission tomography (PET; AV45 > 1.11) or CSF (Aβ < 192 pg/ml) [13]. Furthermore, linear mixed-effects models were employed to test the longitudinal analyses. The models had random intercepts and slopes for time and an unstructured covariance matrix for the random effects. They included the interaction between time (continuous) and genotype (additive model) as a predictor. The “lm”, “nlme”, and “car” packages in R version 3.4.3 were used to perform the above analyses.

### Ethics approval and consent to participate

ADNI was approved by institutional review boards of all participating institutions, and written informed consent was obtained from all participants or their guardians according to the Declaration of Helsinki.

### Availability of data and materials

The datasets used and/or analyzed during the current study are available from the corresponding author on reasonable request.

## Supplementary Material

Supplementary Table 1

Supplementary Table 3

Supplementary Table 6

Supplementary Table 2
