# Supplementary material for: Association of Alzheimer’s disease risk variants on the PICALM gene with PICALM expression, core biomarkers, and feature neurodegeneration
Source: Aging (Albany NY). 2020 Nov 7;12(21):21202–19. doi: 10.18632/aging.103814 (PMC7695360; doi:10.18632/aging.103814)
Supplement: Supplementary Table 1 [file aging-12-103814-s001..docx]

**Supplementary Table 1. Characteristics of studies included in the systematic review.**

| **Author, year [Ref]** | **Ancestry** | **Case** | | | **Control** | | | **Matching variables** | **Adjusted variables** |
| --- | --- | --- | --- | --- | --- | --- | --- | --- | --- |
|  |  | **n** | **age** | **Female (%)** | **N** | **age** | **Female (%)** |  |  |
| Masri, 2019 [1] | unclear | 59 | 78.3 ± 8.0 | 56 | 50 | > 60y | 56 | all analyzed characteristics matched except age | age, gender, body mass index, blood pressure levels, lipid levels, marital status, educational level and *APOE* genotypes |
| Shankarappa, 2017 [4] | mixed | 243 | 67.9 ± 8.7 | 51 | 164 | 66.9 ± 8.7 | 39 | age | *APOE* genotypes |
| Santos-Reboucas, 2017 [5] | unclear | 174 | 77.2 ± 6.4 | 68.90 | 176 | 70.7 ± 6.04 | 73.44 | none | none |
| Moreno, 2017 [6] | mixed | 280 | 75.5 ± 7.2 | 76.10 | 357 | 71.0 ± 7.1 | 73.90 | gender | age, gender, *APOE* status, and admixture covariates |
| Wang, 2016 [7] | Han Chinese | 333 | na | na | 334 | na | na | none | *APOE* genotypes |
| Wang, 2016 [7] | Han Chinese | 416 | na | na | 426 | na | na |  |  |
| Rezazadeh, 2016 [8] | mixed | 160 | 76.1 ± 7.8 | 58.80 | 163 | 75.3 ± 6.8 | 58.3 | gender | none |
| Ortega-Rojas, 2016 [9] | unclear | 181 | 74.1 | 66.20 | 181 | 74.1 | 66.2 | age and gender | none |
| Sen, 2015 [11] | unclear | 112 | 75.5±7.6 | 59.80 | 106 | 74.0 ± 5.3 | 50.9 | age, sex, and education | none |
| Belcavello, 2015 [13] | mixed | 82 | 81.2 ± 7.5 | 65.90 | 161 | 79.4 ± 7.9 | 73.3 | age and gender | age, gender, education, and *APOE4* status |
| Jiang, 2014 [17] | Han Chinese | 1133 | 79.9 ± 8.1 | 59 | 1159 | 74.5 ±6.3 | 55.2 | age and gender | age, gender, and *APOE4* status |
| Yu, 2011 [33] | Han Chinese | 266 | 77.0 ± 6.6 | 54 | 343 | 77 ± 5.9 | 52 | none | age, sex, and *APOE4* |
| Gharesouran, 2014 [19] | Azeri Turkish (mixed) | 160 | 76.1 ± 7.8 | 58.80 | 163 | 75.3 ± 6.8 | 58.3 | gender | none |
| Carrasquillo, 2014 [20] | mixed | 54 | 80.0 ± 10.0 | 76 | 2523 | 81.8 ± 6.1 | 56.7 | none | age, sex, and APOE4 |
| Beecham, 2014 [21] | mixed | 4173 | unclear | 49 to 76 | 1031 | 71 to 89 | 37 to 65 | none | none |
| Miyashita, 2013 [22] | Japanese | 1008 | unclear | 72 | 1016 | 77.0 ± 5.9 | 57 | none | none |
|  | Korean | 339 | 73.7 ± 9.5 | 72 | 1129 | 71.0 ± 4.9 | 49 | none | none |
| Klimkowicz-Mrowiec, 2013 [24] | Caucasian | 253 | 73.9 ± 5.2 | 68.4 | 240 | 73.8 ± 6.9 | 57.5 | age | none |
| Chung, 2013 [25] | Korean | 290 | 74.9 ± 9.1 | 66.9 | 554 | 64.7 ± 9.3 | 51.8 | none | none |
| Rosenthal, 2012 [26] | Caucasian | 1291 | unclear | 66 | 958 | 74.1 + 6.2 | 60 | none | age, sex, APOE4, and the first four principal components |
| Kamboh, 2012 [28] | Caucasian | 1348 | unclear | 65.6 | 1359 | 74.7 ± 6.5 | 60.8 | none | age, sex, APOE4, and the first four principal components |
| Ohara, 2012 [27] | Japanese | 825 | 83.2 ± 6.5 | 77.1 | 2934 | 60.2 ± 11.5 | 56 | none | age, sex, APOE4, and the first four principal components |
| Kamboh, 2012 [29] | Caucasian | 1440 | unclear | 65.6 | 1000 | 74.1 ± 6.2 | 59.8 | none | age, sex, APOE4, and the first four principal components |
|  | unclear | 844 | unclear | 57.2 | 1255 | 73.2 ± 4.4 | 51.7 | none | age, sex, APOE4, and the first four principal components |
|  | mixed | 188 | unclear | 44.6 | 193 | 78.6 ± 5.3 | 46.3 | none | age, sex, APOE4, and the first four principal components |
|  | unclear | 1186 | unclear | na | 1135 | 74.0 ± 8.3 | 61 | none | age, sex, APOE4, and the first four principal components |
|  | Caucasian | 509 | unclear | na | 753 | na | na | none | age, sex, APOE4, and the first four principal components |
| Ferrari, 2012 [30] | mixed (Caucasian 92.4%) | 342 | 76.8 ± 8.6 | 59.1 | 277 | 70.2 ± 8.6 | 64.6 | none | none |
| Chen, 2012 [32] | Chinese | 462 | unclear | na | 350 | na | na | none | age, sex, and APOE4 |
| Lee, 2011 [37] | Caribbean Hispanic | 549 | 78.87 ± 6.4 for total | 69.7 | 544 | 78.9 ± 6.4 | 69.7 | none | age, sex, education, and population stratification |
| Naj, 2011 [35] | Caucasian | 566 | 83.9 ± 4.8 | 63 | 1696 | 81.1 ± 6.0 | 56 | none | none |
|  |  | 1566 | 72.5 ± 7.1 | 54 | 515 | 75.0 ± 8.0 | 59 | none | none |
|  |  | 738 | 73.2 ± 7.1 | 51 | 160 | 75.7 ± 7.9 | 69 | none | none |
|  |  | 268 | 75.3 ± 7.2 | 42 | 173 | 78.6 ± 5.5 | 40 | none | none |
|  |  | 669 | 74.6 ± 6.2 | 57 | 713 | 74.2 ± 7.0 | 64 | none | none |
|  |  | 1186 | 74.1 ± 7.8 | 64 | 1135 | 74.0 ± 8.3 | 61 | none | none |
|  |  | 509 | 71.2 ± 6.5 | 64 | 753 | 72.0 ± 7.2 | 58 | age and gender | none |
|  |  | 1811 | 73.6 ± 6.7 | 65 | 1575 | 74.0 ± 8.5 | 60 | none | none |
|  |  | 132 | 86.1 ± 5.5 | 61 | 153 | 83.9 ± 7.6 | 55 | none | none |
|  |  | 864 | 74.9 ± 7.2 | 73 | 493 | 80.2 ± 8.7 | 38 | none | none |
|  |  | 897 | 75.0 ± 8.5 | 55 | 588 | 75.3 ± 9.8 | 63 | none | none |
|  |  | 728 | unclear | 58 | 1173 | 73.3 ± 4.4 | 51 | none | none |
|  |  | 296 | 85.6 ± 6.3 | 70 | 776 | 82.0 ± 7.0 | 72 | none | none |
|  |  | 1271 | 72.9 ± 6.4 | 63 | 841 | 75.4 ± 6.1 | 63 | none | none |
|  |  | 339 | unclear | 57 | 187 | 76.9 ± 8.4 | 60 | none | none |
| Lambert, 2011 [38] | Caucasian | 561 | unclear | na | 521 | na | na | none | age, gender, disease status, and (when necessary) center |
|  |  | 1460 | unclear | na | 1257 | na | na | none |  |
|  |  | 723 | unclear | na | 819 | na | na | none |  |
| Seshadri, 2010 [40] | only data for Spanish was used for meta | 1140 | unclear | na | 1209 | na | na | none | age, gender, and APOE4 status |
| Jun, 2010 [41] | Caucasian | 1595 | unclear | na | 553 | 77 ± 8.7 | na | none | age, gender, and APOE4 status |
|  |  | 286 | unclear | na | 195 | 78 ± 5.4 | na | none |  |
|  |  | 127 | unclear | na | 105 | 76 ± 7.8 | na | none |  |
|  |  | 197 | unclear | na | 2392 | 73 ± 7.5 | na | none |  |
|  |  | 1170 | unclear | na | 1169 | 74 ± 7.6 | na | age and gender |  |
|  |  | 560 | unclear | na | 790 | 72 ± 7.1 | na | none |  |
|  |  | 993 | unclear | na | 884 | 76 ± 8.4 | na | none |  |
|  |  | 187 | unclear | na | 429 | 86 ± 7.2 | na | none |  |
|  |  | 820 | unclear | na | 517 | 83 ± 8.9 | na | none |  |
|  | African American | 61 | unclear | na | 63 | 76 ± 6.2 | na | none | none |
|  |  | 221 | unclear | na | 186 | 78 ± 6.6 | na | none | none |
|  |  | 180 | unclear | na | 200 | 71 ± 10 | na | none | none |
|  | Arab | 124 | unclear | na | 142 | 72 ± 6.0 | na | none | none |
|  | Hispanic | 549 | unclear | na | 544 | 79 ± 6.4 | na | none | none |
| Corneveaux, 2010 [42] | Caucasian | 1019 | unclear | 64 | 591 | na | 52% | none | none |
| Carrasquillo, 2010, USA [20] | Caucasian | 1819 | unclear | na | 2576 | na | na | none | age, gender, and APOE4 status |
| Harold, 2009 [44] | USA plus Europe | 3941 | 78.6 | 62.7 | 7848 | na | 53.4 | none | none |
|  |  | 2023 | 78.2 | 66.2 | 2340 | 69.8 | 59.1 | none | none |
| Ding, 2012 [31] | Chinese | 54 | 81.2 ± 5.3 | 63 | 216 | 80.4 ± 4.9 | 63 | age and gender | age, gender, education, tea, and APOE4 status |
| Xiao, 2015 Li, 2011 [10,36] | Han Chinese | 232 | 72.0 ± 9.5 | 51.3 | 373 | 72.5 ± 5.9 | 52.9 | gender | age, gender, and APOE4 status |
| Li, 2011 [36] | Han Chinese | 380 | 69.5 ± 10 | 59.3 | 307 | 68.6 ± 9.6 | 62.1 | age and gender | none |
| Wang, 2014 [14] | Han Chinese | 250 | 77.4 ± 8.4 | 36.8 | 438 | 70.2 ± 7.5 | 30.6 | none | none |
| Hui, 2014 [18] | Han Chinese | 248 | 77.4 ± 8.4 | 37.1 | 408 | 70.2 ± 7.5 | 32.8 | age and gender | none |
| Liu, 2014 [16] | Han Chinese | 239 | 74.6 ± 5.9 | 62.3 | 207 | 72.0 ± 5.5 | 44.4 | none | none |
| Jiao, 2015 [12] | Han Chinese | 229 | 75.2 ± 5.0 | 56.9 | 318 | 71.6 ± 2.5 | 52.2 | none | age and gender |
| Piaceri, 2011 [34] | Caucasian | 349 | 74.04 ± 6.1 | 65.6 | 359 | 74.5 ± 6.2 | 55.9 | age and gender | none |
| Omoumi, 2014 [15] | unclear | 428 | 78.2 ± 7.2 | na | 524 | 76.0 ± 6.5 | na | none | APOE and age |
| Seripa, 2017 [3] | Caucasian | 520 | 74.7 ± 8.5 | 66.2 | 569 | 71.9 ± 11.8 | 54 | none | none |
| Kunkle, 2019 [2] | majorly Caucasian | 14428 | na | 59.3 | 14562 | 76.2 ± 9.9 | 59.3 | na | na |
|  |  | 2137 | na | 67.3 | 13474 | 76.7 ± 8.2 | 55.8 | na | na |
|  |  | 2240 | na | 65 | 6631 | 78.9 ± 7.0 | 60.6 | na | na |
|  |  | 3177 | na | 64 | 7277 | 51.0 ± 0.1 | 51.8 | na | na |
|  |  | 878 | na | 66.1 | 661 | 65.7 ± 14.3 | 59.5 | na | na |
|  |  | 422 | na | 68 | 562 | 69.1 ± 6.2 | 59.3 | na | na |
|  |  | 972 | na | 63.9 | 2378 | 69.5 ± 10.1 | 53.1 | na | na |
|  |  | 256 | na | 63.3 | 229 | 49.3 ± 16.4 | 34.1 | na | na |
|  |  | 125 | na | 68 | 100 | 74.4 ± 6.5 | 69 | na | na |
|  |  | 1729 | na | 66.5 | 720 | 70.0 ± 10.4 | 55.7 | na | na |
|  |  | 2121 | na | 66.3 | 1921 | 70.2 ± 10.8 | 55.3 | na | na |
|  |  | 797 | na | 61.7 | 1506 | 70.6 ± 8.7 | 62.8 | na | na |
|  |  | 490 | na | 67.6 | 1066 | 73.8 ± 6.5 | 29.2 | na | na |
|  |  | 572 | na | 61.9 | 1340 | 79.3 ± 6.8 | 54 | na | na |
|  |  | 932 | na | 71 | 1813 | 54.7 ± 12.1 | 68.4 | na | na |
|  |  | 1902 | na | 64.80 | 1047 | 73.9 ± 12.9 | 57.8 | na | na |
|  |  | 514 | na | 51.30 | 790 | 72.3 ± 7.7 | 63.6 | na | na |
|  |  | 1582 | na | 74.60 | 3086 | 54.0 ± 14.0 | 48 | na | na |
| Hollingworth, 2011 [39] | unclear | 3941 | 78.6 | 62.70 | 7848 | 55.6 | 65.8 | na | na |
|  | French Caucasian | 2025 | 73.7 | 66 | 5328 | 73.8 | 61 | na | na |
|  | mixed | 151 | 76.6 | 47 | 177 | 78 | 44.6 | na | na |
|  | unclear | 571 | 81 | 52 | 332 | 80 | 63 | na | na |

References

1. Masri I, Salami A, El Shamieh S, Bissar-Tadmouri N. rs3851179G>A in PICALM is protective against Alzheimer’s disease in five different countries surrounding the mediterranean. Curr Aging Sci. 2019. [Epub ahead of print].

<https://doi.org/10.2174/1874609812666191019143237> PMID:[31648652](https://pubmed.ncbi.nlm.nih.gov/31648652)

2. Kunkle BW, Grenier-Boley B, Sims R, Bis JC, Damotte V, Naj AC, Boland A, Vronskaya M, van der Lee SJ, Amlie-Wolf A, Bellenguez C, Frizatti A, Chouraki V, et al, Alzheimer Disease Genetics Consortium (ADGC),, European Alzheimer’s Disease Initiative (EADI), Cohorts for Heart and Aging Research in Genomic Epidemiology Consortium (CHARGE), and Genetic and Environmental Risk in AD/Defining Genetic, Polygenic and Environmental Risk for Alzheimer’s Disease Consortium (GERAD/PERADES). Genetic meta-analysis of diagnosed Alzheimer’s disease identifies new risk loci and implicates Aβ, tau, immunity and lipid processing. Nat Genet. 2019; 51:414–30.

<https://doi.org/10.1038/s41588-019-0358-2> PMID:[30820047](https://pubmed.ncbi.nlm.nih.gov/30820047)

3. Seripa D, Panza F, Paroni G, D’Onofrio G, Bisceglia P, Gravina C, Urbano M, Lozupone M, Solfrizzi V, Bizzarro A, Boccardi V, Piccininni C, Daniele A, et al. Role of CLU, PICALM, and TNK1 genotypes in aging with and without Alzheimer’s disease. Mol Neurobiol. 2018; 55:4333–44.

<https://doi.org/10.1007/s12035-017-0547-x> PMID:[28631188](https://pubmed.ncbi.nlm.nih.gov/28631188)

4. Shankarappa BM, Kota LN, Purushottam M, Nagpal K, Mukherjee O, Viswanath B, Varghese M, Bharath S, Jain S. Effect of CLU and PICALM polymorphisms on AD risk: a study from south India. Asian J Psychiatr. 2017; 27:7–11.

<https://doi.org/10.1016/j.ajp.2016.12.017> PMID:[28558900](https://pubmed.ncbi.nlm.nih.gov/28558900)

5. Santos-Rebouças CB, Gonçalves AP, Dos Santos JM, Abdala BB, Motta LB, Laks J, de Borges MB, de Rosso AL, Pereira JS, Nicaretta DH, Pimentel MM. Rs3851179 polymorphism at 5’ to the PICALM gene is associated with Alzheimer and Parkinson diseases in Brazilian population. Neuromolecular Med. 2017; 19:293–99.

<https://doi.org/10.1007/s12017-017-8444-z> PMID:[28567584](https://pubmed.ncbi.nlm.nih.gov/28567584)

6. Moreno DJ, Ruiz S, Ríos Á, Lopera F, Ostos H, Via M, Bedoya G. Association of GWAS top genes with late-onset Alzheimer’s disease in Colombian population. Am J Alzheimers Dis Other Demen. 2017; 32:27–35.

<https://doi.org/10.1177/1533317516679303> PMID:[28084078](https://pubmed.ncbi.nlm.nih.gov/28084078)

7. Wang HZ, Bi R, Hu QX, Xiang Q, Zhang C, Zhang DF, Zhang W, Ma X, Guo W, Deng W, Zhao L, Ni P, Li M, et al. Validating GWAS-identified risk loci for Alzheimer’s disease in Han Chinese populations. Mol Neurobiol. 2016; 53:379–90.

<https://doi.org/10.1007/s12035-014-9015-z> PMID:[25452228](https://pubmed.ncbi.nlm.nih.gov/25452228)

8. Rezazadeh M, Khorrami A, Yeghaneh T, Talebi M, Kiani SJ, Heshmati Y, Gharesouran J. Genetic factors affecting late-onset Alzheimer’s disease susceptibility. Neuromolecular Med. 2016; 18:37–49.

<https://doi.org/10.1007/s12017-015-8376-4> PMID:[26553058](https://pubmed.ncbi.nlm.nih.gov/26553058)

9. Ortega-Rojas J, Morales L, Guerrero E, Arboleda-Bustos CE, Mejia A, Forero D, Lopez L, Pardo R, Arboleda G, Yunis J, Arboleda H. Association analysis of polymorphisms in TOMM40, CR1, PVRL2, SORL1, PICALM, and 14q32.13 regions in Colombian Alzheimer disease patients. Alzheimer Dis Assoc Disord. 2016; 30:305–09.

<https://doi.org/10.1097/WAD.0000000000000142> PMID:[27023435](https://pubmed.ncbi.nlm.nih.gov/27023435)

10. Xiao Q, Liu ZJ, Tao S, Sun YM, Jiang D, Li HL, Chen H, Liu X, Lapin B, Wang CH, Zheng SL, Xu J, Wu ZY. Risk prediction for sporadic Alzheimer’s disease using genetic risk score in the Han Chinese population. Oncotarget. 2015; 6:36955–64.

<https://doi.org/10.18632/oncotarget.6271> PMID:[26543236](https://pubmed.ncbi.nlm.nih.gov/26543236)

11. Sen A, Arslan M, Erdal ME, Ay OI, Yilmaz SG, Kurt E, Arpaci B. Lack of associations between clu and picalm gene polymorphisms and Alzheimer’s disease in a Turkish population. Ideggyogy Sz. 2015; 68:113–20.

PMID:[26434199](https://pubmed.ncbi.nlm.nih.gov/26434199)

12. Jiao B, Liu X, Zhou L, Wang MH, Zhou Y, Xiao T, Zhang W, Sun R, Waye MM, Tang B, Shen L. Polygenic analysis of late-onset Alzheimer’s disease from mainland China. PLoS One. 2015; 10:e0144898.

<https://doi.org/10.1371/journal.pone.0144898> PMID:[26680604](https://pubmed.ncbi.nlm.nih.gov/26680604)

13. Belcavello L, Camporez D, Almeida LD, Morelato RL, Batitucci MC, de Paula F. Association of MTHFR and PICALM polymorphisms with Alzheimer’s disease. Mol Biol Rep. 2015; 42:611–16.

<https://doi.org/10.1007/s11033-014-3806-1> PMID:[25359311](https://pubmed.ncbi.nlm.nih.gov/25359311)

14. Wang NN ZZ, Zhang YH. The molecular epidemiology research of susceptibility genes of Alzheimer’s disease in Northern Chinese population. Chinese master dissertation. 2014.

15. Omoumi A, Fok A, Greenwood T, Sadovnick AD, Feldman HH, Hsiung GY. Evaluation of late-onset Alzheimer disease genetic susceptibility risks in a Canadian population. Neurobiol Aging. 2014; 35:936.e5–12.

<https://doi.org/10.1016/j.neurobiolaging.2013.09.025> PMID:[24176626](https://pubmed.ncbi.nlm.nih.gov/24176626)

16. Liu XY SL. Association analysis of late-onset Alzheimer’s disease and susceptibility genes in Chinese Han population. Chinese master dissertation. 2014.

17. Jiang T, Yu JT, Tan MS, Wang HF, Wang YL, Zhu XC, Zhang W, Tan L. Genetic variation in PICALM and Alzheimer’s disease risk in Han Chinese. Neurobiol Aging. 2014; 35:934.e1–3.

<https://doi.org/10.1016/j.neurobiolaging.2013.09.014> PMID:[24095218](https://pubmed.ncbi.nlm.nih.gov/24095218)

18. Hui J YZ, Zhang YH. Association Analysis of Eight Gene Variations with Alzheimer’s isease Susceptibility in Northen Chinese populations. Chinese doctoral dissertation. 2014.

19. Gharesouran J, Rezazadeh M, Khorrami A, Ghojazadeh M, Talebi M. Genetic evidence for the involvement of variants at APOE, BIN1, CR1, and PICALM loci in risk of late-onset Alzheimer’s disease and evaluation for interactions with APOE genotypes. J Mol Neurosci. 2014; 54:780–86.

<https://doi.org/10.1007/s12031-014-0377-5> PMID:[25022885](https://pubmed.ncbi.nlm.nih.gov/25022885)

20. Carrasquillo MM, Khan Qu, Murray ME, Krishnan S, Aakre J, Pankratz VS, Nguyen T, Ma L, Bisceglio G, Petersen RC, Younkin SG, Dickson DW, Boeve BF, et al. Late-onset Alzheimer disease genetic variants in posterior cortical atrophy and posterior AD. Neurology. 2014; 82:1455–62.

<https://doi.org/10.1212/WNL.0000000000000335> PMID:[24670887](https://pubmed.ncbi.nlm.nih.gov/24670887)

21. Beecham GW, Hamilton K, Naj AC, Martin ER, Huentelman M, Myers AJ, Corneveaux JJ, Hardy J, Vonsattel JP, Younkin SG, Bennett DA, De Jager PL, Larson EB, et al, and Alzheimer’s Disease Genetics Consortium (ADGC). Genome-wide association meta-analysis of neuropathologic features of Alzheimer’s disease and related dementias. PLoS Genet. 2014; 10:e1004606.

<https://doi.org/10.1371/journal.pgen.1004606> PMID:[25188341](https://pubmed.ncbi.nlm.nih.gov/25188341)

22. Miyashita A, Koike A, Jun G, Wang LS, Takahashi S, Matsubara E, Kawarabayashi T, Shoji M, Tomita N, Arai H, Asada T, Harigaya Y, Ikeda M, et al, and Alzheimer Disease Genetics Consortium. SORL1 is genetically associated with late-onset Alzheimer’s disease in Japanese, Koreans and Caucasians. PLoS One. 2013; 8:e58618.

<https://doi.org/10.1371/journal.pone.0058618> PMID:[23565137](https://pubmed.ncbi.nlm.nih.gov/23565137)

23. Lambert JC, Ibrahim-Verbaas CA, Harold D, Naj AC, Sims R, Bellenguez C, DeStafano AL, Bis JC, Beecham GW, Grenier-Boley B, Russo G, Thorton-Wells TA, Jones N, et al, European Alzheimer’s Disease Initiative (EADI), Genetic and Environmental Risk in Alzheimer’s Disease, Alzheimer’s Disease Genetic Consortium, and Cohorts for Heart and Aging Research in Genomic Epidemiology. Meta-analysis of 74,046 individuals identifies 11 new susceptibility loci for Alzheimer’s disease. Nat Genet. 2013; 45:1452–58.

<https://doi.org/10.1038/ng.2802> PMID:[24162737](https://pubmed.ncbi.nlm.nih.gov/24162737)

24. Klimkowicz-Mrowiec A, Sado M, Dziubek A, Dziedzic T, Pera J, Szczudlik A, Slowik A. Lack of association of CR1, PICALM and CLU gene polymorphisms with Alzheimer disease in a polish population. Neurol Neurochir Pol. 2013; 47:157–60.

<https://doi.org/10.5114/ninp.2013.33825> PMID:[23650005](https://pubmed.ncbi.nlm.nih.gov/23650005)

25. Chung SJ, Lee JH, Kim SY, You S, Kim MJ, Lee JY, Koh J. Association of GWAS top hits with late-onset Alzheimer disease in Korean population. Alzheimer Dis Assoc Disord. 2013; 27:250–57.

<https://doi.org/10.1097/WAD.0b013e31826d7281> PMID:[22975751](https://pubmed.ncbi.nlm.nih.gov/22975751)

26. Rosenthal SL, Wang X, Demirci FY, Barmada MM, Ganguli M, Lopez OL, Kamboh MI. Beta-amyloid toxicity modifier genes and the risk of Alzheimer’s disease. Am J Neurodegener Dis. 2012; 1:191–98.

PMID:[22984654](https://pubmed.ncbi.nlm.nih.gov/22984654)

27. Ohara T, Ninomiya T, Hirakawa Y, Ashikawa K, Monji A, Kiyohara Y, Kanba S, Kubo M. Association study of susceptibility genes for late-onset Alzheimer’s disease in the Japanese population. Psychiatr Genet. 2012; 22:290–93.

<https://doi.org/10.1097/YPG.0b013e3283586215> PMID:[22935915](https://pubmed.ncbi.nlm.nih.gov/22935915)

28. Kamboh MI, Minster RL, Demirci FY, Ganguli M, Dekosky ST, Lopez OL, Barmada MM. Association of CLU and PICALM variants with Alzheimer’s disease. Neurobiol Aging. 2012; 33:518–21.

<https://doi.org/10.1016/j.neurobiolaging.2010.04.015> PMID:[20570404](https://pubmed.ncbi.nlm.nih.gov/20570404)

29. Kamboh MI, Demirci FY, Wang X, Minster RL, Carrasquillo MM, Pankratz VS, Younkin SG, Saykin AJ, Jun G, Baldwin C, Logue MW, Buros J, Farrer L, et al, and Alzheimer’s Disease Neuroimaging Initiative. Genome-wide association study of Alzheimer’s disease. Transl Psychiatry. 2012; 2:e117.

<https://doi.org/10.1038/tp.2012.45> PMID:[22832961](https://pubmed.ncbi.nlm.nih.gov/22832961)

30. Ferrari R, Moreno JH, Minhajuddin AT, O’Bryant SE, Reisch JS, Barber RC, Momeni P. Implication of common and disease specific variants in CLU, CR1, and PICALM. Neurobiol Aging. 2012; 33:1846.e7–18.

<https://doi.org/10.1016/j.neurobiolaging.2012.01.110> PMID:[22402018](https://pubmed.ncbi.nlm.nih.gov/22402018)

31. Ding D HZ. Population-based prevalence survey and genetic epidemiology of cognitive impairment among elderly. Chinese doctoral dissertation. 2012.

32. Chen LH, Kao PY, Fan YH, Ho DT, Chan CS, Yik PY, Ha JC, Chu LW, Song YQ. Polymorphisms of CR1, CLU and PICALM confer susceptibility of Alzheimer’s disease in a southern Chinese population. Neurobiol Aging. 2012; 33:210.e1–7.

<https://doi.org/10.1016/j.neurobiolaging.2011.09.016> PMID:[22015308](https://pubmed.ncbi.nlm.nih.gov/22015308)

33. Yu JT, Song JH, Ma T, Zhang W, Yu NN, Xuan SY, Tan L. Genetic association of PICALM polymorphisms with Alzheimer’s disease in Han Chinese. J Neurol Sci. 2011; 300:78–80.

<https://doi.org/10.1016/j.jns.2010.09.027> PMID:[20951388](https://pubmed.ncbi.nlm.nih.gov/20951388)

34. Piaceri I, Bagnoli S, Lucenteforte E, Mancuso M, Tedde A, Siciliano G, Piacentini S, Bracco L, Sorbi S, Nacmias B. Implication of a genetic variant at PICALM in Alzheimer’s disease patients and centenarians. J Alzheimers Dis. 2011; 24:409–13.

<https://doi.org/10.3233/JAD-2011-101791> PMID:[21297266](https://pubmed.ncbi.nlm.nih.gov/21297266)

35. Naj AC, Jun G, Beecham GW, Wang LS, Vardarajan BN, Buros J, Gallins PJ, Buxbaum JD, Jarvik GP, Crane PK, Larson EB, Bird TD, Boeve BF, et al. Common variants at MS4A4/MS4A6E, CD2AP, CD33 and EPHA1 are associated with late-onset Alzheimer’s disease. Nat Genet. 2011; 43:436–41.

<https://doi.org/10.1038/ng.801> PMID:[21460841](https://pubmed.ncbi.nlm.nih.gov/21460841)

36. Li HL, Shi SS, Guo QH, Ni W, Dong Y, Liu Y, Sun YM, Bei-Wang, Lu SJ, Hong Z, Wu ZY. PICALM and CR1 variants are not associated with sporadic Alzheimer’s disease in Chinese patients. J Alzheimers Dis. 2011; 25:111–17.

<https://doi.org/10.3233/JAD-2011-101917> PMID:[21358043](https://pubmed.ncbi.nlm.nih.gov/21358043)

37. Lee JH, Cheng R, Barral S, Reitz C, Medrano M, Lantigua R, Jiménez-Velazquez IZ, Rogaeva E, St George-Hyslop PH, Mayeux R. Identification of novel loci for Alzheimer disease and replication of CLU, PICALM, and BIN1 in Caribbean Hispanic individuals. Arch Neurol. 2011; 68:320–28.

<https://doi.org/10.1001/archneurol.2010.292> PMID:[21059989](https://pubmed.ncbi.nlm.nih.gov/21059989)

38. Lambert JC, Zelenika D, Hiltunen M, Chouraki V, Combarros O, Bullido MJ, Tognoni G, Fiévet N, Boland A, Arosio B, Coto E, Del Zompo M, Mateo I, et al. Evidence of the association of BIN1 and PICALM with the AD risk in contrasting European populations. Neurobiol Aging. 2011; 32:756.e11–15.

<https://doi.org/10.1016/j.neurobiolaging.2010.11.022> PMID:[21220176](https://pubmed.ncbi.nlm.nih.gov/21220176)

39. Hollingworth P, Harold D, Sims R, Gerrish A, Lambert JC, Carrasquillo MM, Abraham R, Hamshere ML, Pahwa JS, Moskvina V, Dowzell K, Jones N, Stretton A, et al, Alzheimer’s Disease Neuroimaging Initiative, CHARGE consortium, and EADI1 consortium. Common variants at ABCA7, MS4A6A/MS4A4E, EPHA1, CD33 and CD2AP are associated with Alzheimer’s disease. Nat Genet. 2011; 43:429–35.

<https://doi.org/10.1038/ng.803> PMID:[21460840](https://pubmed.ncbi.nlm.nih.gov/21460840)

40. Seshadri S, Fitzpatrick AL, Ikram MA, DeStefano AL, Gudnason V, Boada M, Bis JC, Smith AV, Carassquillo MM, Lambert JC, Harold D, Schrijvers EM, Ramirez-Lorca R, et al, CHARGE Consortium, GERAD1 Consortium, and EADI1 Consortium. Genome-wide analysis of genetic loci associated with Alzheimer disease. JAMA. 2010; 303:1832–40.

<https://doi.org/10.1001/jama.2010.574> PMID:[20460622](https://pubmed.ncbi.nlm.nih.gov/20460622)

41. Jun G, Naj AC, Beecham GW, Wang LS, Buros J, Gallins PJ, Buxbaum JD, Ertekin-Taner N, Fallin MD, Friedland R, Inzelberg R, Kramer P, Rogaeva E, et al, and Alzheimer’s Disease Genetics Consortium. Meta-analysis confirms CR1, CLU, and PICALM as Alzheimer disease risk loci and reveals interactions with APOE genotypes. Arch Neurol. 2010; 67:1473–84.

<https://doi.org/10.1001/archneurol.2010.201> PMID:[20697030](https://pubmed.ncbi.nlm.nih.gov/20697030)

42. Corneveaux JJ, Myers AJ, Allen AN, Pruzin JJ, Ramirez M, Engel A, Nalls MA, Chen K, Lee W, Chewning K, Villa SE, Meechoovet HB, Gerber JD, et al. Association of CR1, CLU and PICALM with Alzheimer’s disease in a cohort of clinically characterized and neuropathologically verified individuals. Hum Mol Genet. 2010; 19:3295–301.

<https://doi.org/10.1093/hmg/ddq221> PMID:[20534741](https://pubmed.ncbi.nlm.nih.gov/20534741)

43. Carrasquillo MM, Belbin O, Hunter TA, Ma L, Bisceglio GD, Zou F, Crook JE, Pankratz VS, Dickson DW, Graff-Radford NR, Petersen RC, Morgan K, Younkin SG. Replication of CLU, CR1, and PICALM associations with Alzheimer disease. Arch Neurol. 2010; 67:961–64.

<https://doi.org/10.1001/archneurol.2010.147> PMID:[20554627](https://pubmed.ncbi.nlm.nih.gov/20554627)

44. Harold D, Abraham R, Hollingworth P, Sims R, Gerrish A, Hamshere ML, Pahwa JS, Moskvina V, Dowzell K, Williams A, Jones N, Thomas C, Stretton A, et al. Genome-wide association study identifies variants at CLU and PICALM associated with Alzheimer’s disease. Nat Genet. 2009; 41:1088–93.

<https://doi.org/10.1038/ng.440> PMID:[19734902](https://pubmed.ncbi.nlm.nih.gov/19734902)
