# Supplementary material for: Association of Alzheimer’s disease risk variants on the PICALM gene with PICALM expression, core biomarkers, and feature neurodegeneration
Source: Aging (Albany NY). 2020 Nov 7;12(21):21202–19. doi: 10.18632/aging.103814 (PMC7695360; doi:10.18632/aging.103814)
Supplement: Supplementary Table 3 [file aging-12-103814-s002..pdf]

SUPPLEMENTARY METHODS

APPENDIX 1 METHODS FOR SYSTEMATIC REVIEW AND META-ANALYSIS

Search strategy and selection criteria

We followed the recommendations by the Preferred Reporting Items for Systematic Reviews and Meta-Analyses (PRISMA) 2009 guidelines [1, 2]. Electronic databases published in English (PubMed and EMBASE) and Chinese (CNKI) using terms "PICALM" and "CALM", till Jan 11, 2020. Bibliographies of relevant original studies and systematic reviews were hand-searched in case of omission. The inclusion criteria were as follows: (i) the study explored the associations of PICALM gene with AD risk, and (ii) the study provided the risk estimates or the raw data that can be used to calculate these numbers. Studies were excluded if they met any of the following criteria: 1) risk estimate is not accessible or could not be calculated; 2) only abstracts were available, 3) editorials or comments. Literature selection was performed by two experienced investigators (WX and CCT) and any disagreements on inclusion were resolved by consensus and arbitration within the review team (WX, CCT, and LT).

Data extraction

Pre-designed templates were used to extract the data, including first author, publication year, country/region, ancestry, sample size (case and control group), characteristics of case group (age at exam, age of onset, female percentage, source, AD type, diagnosis criteria, and if autopsy-confirmed or not) and control group (age, female percentage, source, and neuropsychological evaluation), matching variables, adjusted variables, genotyping method, identified *PICALM* loci associated with AD risk, and the multivariable-adjusted risk estimates. If any data mentioned above were unavailable, we attempted to obtain them via contacting the corresponding authors. The data extraction was performed by two experienced investigators (WX and CCT) and any discrepancies were addressed by negotiation with the third reviewer (LT).

Assessment of the study quality and credibility of meta-analyses

An evolving Newcastle-Ottawa Quality Assessment Scale (NOS) for observational case-control studies was employed to evaluate the quality of eligible studies. The total score of NOS was regarded here as a proxy to assess the overall risk of bias for each single study.

Statistical analyses

The multivariable-adjusted risk estimates and 95% confidence intervals (CI) were log-transformed and pooled using random models (DerSimonian-Laird method) [3]. Heterogeneity was assessed by Q test and quantified by the I^2 metric . All analyses were conducted according to ethnicity. The source of heterogeneity was explored via sensitivity analyses, meta-regression (if $N \geq 10$), and subgroup analyses. The robustness of the results was examined by excluding those rated as at a higher risk of bias. Publication bias was assessed (if $N \geq 10$) following two steps: 1) testing the symmetry of the funnel plot by Egger method ; 2) determining whether any asymmetry was due to publication bias via enhanced-contour funnel plots after the trim-and-fill method . The "metagen", "metabias", and "trimfill" packages in R 3.4.3 software (https://www.r-project.org) were used to perform all these analyses.

REFERENCES

1. Moher D, Liberati A, Tetzlaff J, Altman DG, and PRISMA Group. Preferred reporting items for systematic reviews and meta-analyses: the PRISMA statement. Int J Surg. 2010; 8:336–41. https://doi.org/10.1016/j.ijsu.2010.02.007 PMID:20171303

2. Stroup DF, Berlin JA, Morton SC, Olkin I, Williamson GD, Rennie D, Moher D, Becker BJ, Sipe TA, Thacker SB. Meta-analysis of observational studies in epidemiology: a proposal for reporting. Meta-analysis of observational studies in epidemiology (MOOSE) group. JAMA. 2000; 283:2008–12. https://doi.org/10.1001/jama.283.15.2008 PMID:10789670

3. Higgins JP, Thompson SG, Deeks JJ, Altman DG. Measuring inconsistency in meta-analyses. BMJ. 2003; 327:557–60. https://doi.org/10.1136/bmj.327.7414.557 PMID:12958120
