# Supplementary material for: Association of Alzheimer’s disease risk variants on the PICALM gene with PICALM expression, core biomarkers, and feature neurodegeneration
Source: Aging (Albany NY). 2020 Nov 7;12(21):21202–19. doi: 10.18632/aging.103814 (PMC7695360; doi:10.18632/aging.103814)
Supplement: Supplementary Table 2 [file aging-12-103814-s004..docx]

SUPPLEMENTARY FIGURES


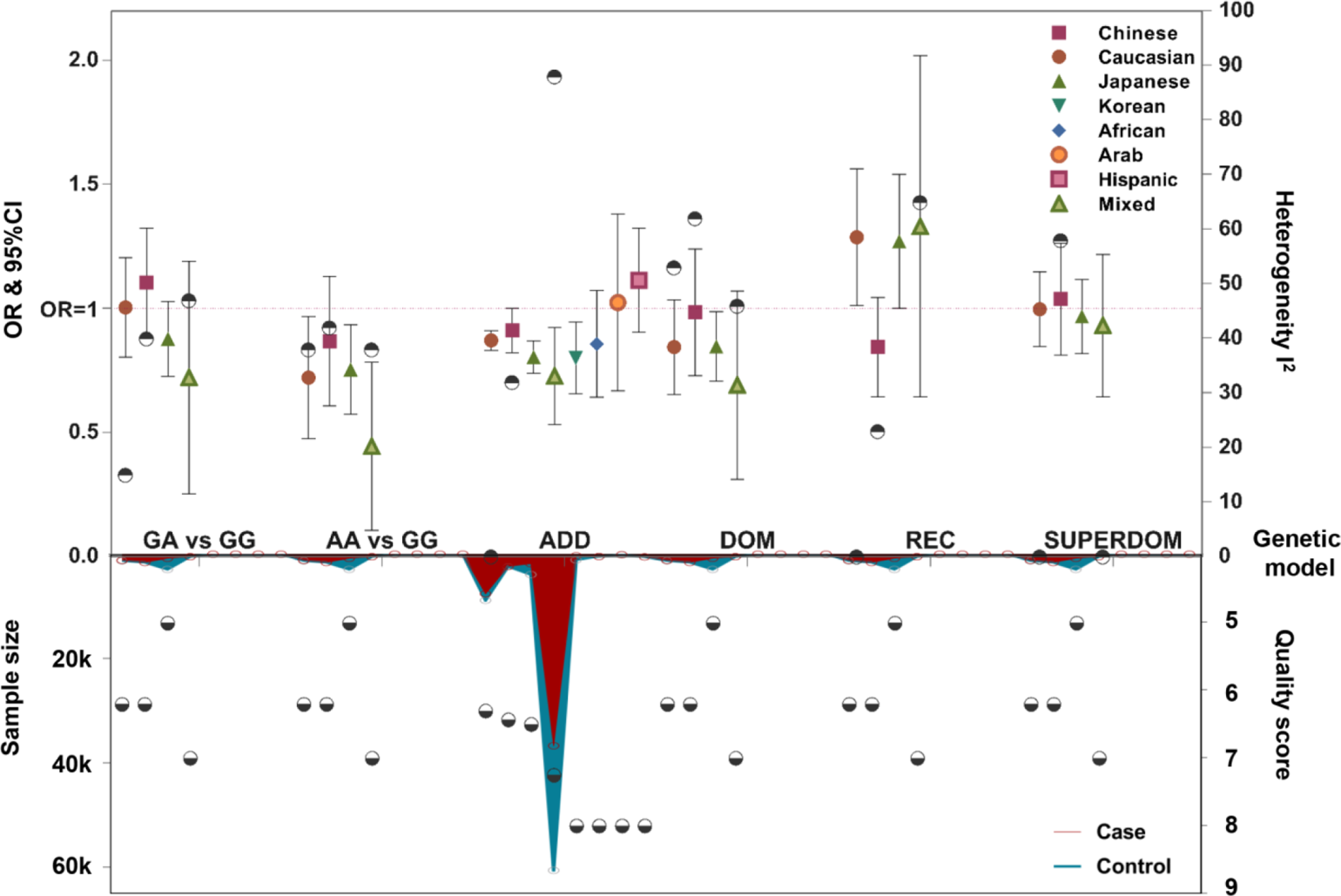


Supplementary Figure 1. Association of rs3851179 with AD risk with different genetic models in different ethnicities. rs3851179 (allele A) was associated with lower AD risk, with the effect size ranging from 9% to 29% in Caucasian (I^2^ = 38%), Chinese (I^2^ = 42%), Japanese, Korean, and population of mixed races (I^2^ = 38%), but not in other races. The association remained significant in Caucasian population for other genetic models, such as REC (AA vs GG+GA), and genotype (AA vs GG).


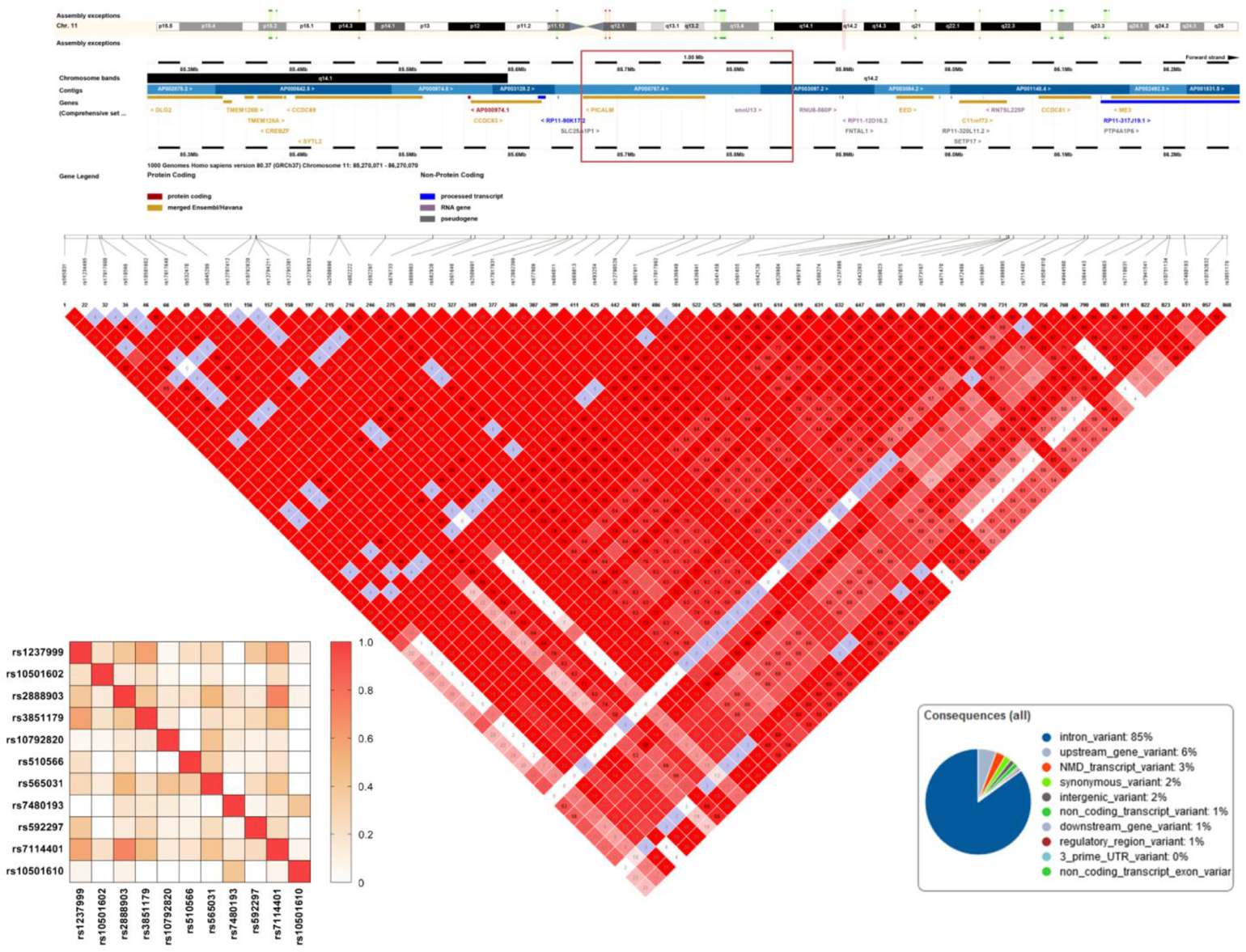


Supplementary Figure 2. Linkage Disequilibrium Analysis revealed eleven tag SNPs. Eleven SNPs were selected by LD analysis, such that these 11 loci could independently capture 100% of all alleles at r^2^ ≥ 0.8.


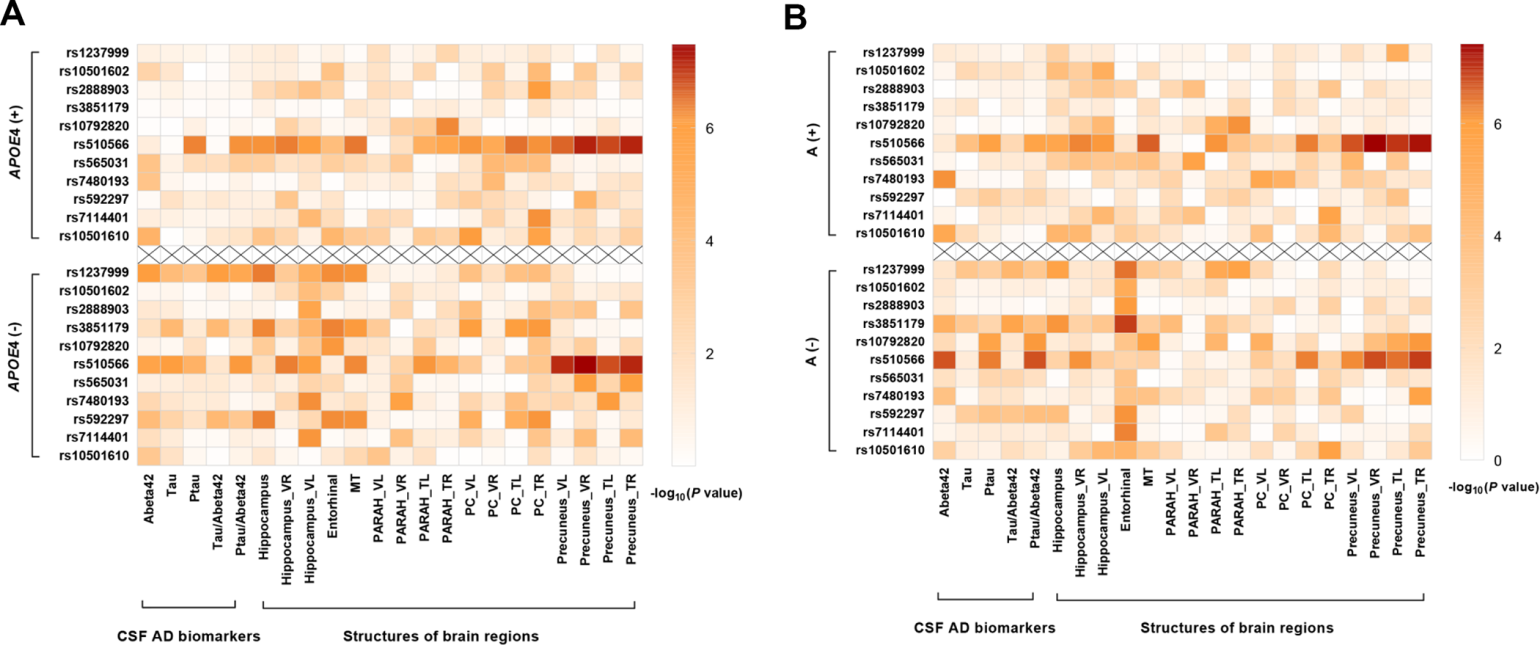


Supplementary Figure 3. Association results of *PICALM* tag loci with AD CSF biomarkers and feature neurodegeneration, stratified by *APOE*4 and amyloid status. The association for rs510566 was not influenced by subgrouping according to APOE4, but remained significant only in A (-) subgroup. The associations with specific loci showed significant trends in APOE4 (-) subgroup, including rs1237999 (p = 0.002 for HIPPO, p = 0.009 for ENTOR, and p = 0.018 for MT), rs592297 (p = 0.003 for HIPPO, p = 0.008 for ENTOR, and p = 0.014 for MT), and rs3851179 (p = 0.005 for HIPPO, p = 0.0037 for ENTOR, p < 0.05 for MT, and p < 0.05 for PC).
